# Supplementary material for: The Effect of Fabry Disease Therapy on Bone Mineral Density
Source: Diseases. 2024 May 13;12(5):102. doi: 10.3390/diseases12050102 (PMC11120203; doi:10.3390/diseases12050102)
Supplement: Supplementary file 1 [file diseases-12-00102-s001.zip › diseases-2921271-supplementary.pdf]

Supplementary Materials

Supplementary Table S1: Fabry specific therapy use in the treated group

Age at commencement of treatment – yrs	39.1 ± 13.4
Fabry specific therapy at baseline	
Agalsidase alfa	20 (52.6)
Agalsidase beta	17 (44.7)
Migalastat	1 (2.6)
Time between commencement of therapy and first DXA – yrs	1.4 [0.4 – 2.4]

Mean ± standard deviation, median [interquartile range] or number (%)

Supplementary Table S2: Longitudinal change in bone density within group

	Within group from linear mixed effects model		
	β-coefficient	95% confidence interval	P-value
**Total hip Z-score**			
Untreated	0.092	0.059, 0.126	**0.000**
Treated	-0.011	-0.042, 0.120	0.490
**Femoral neck Z-score**			
Untreated	0.071	0.032, 0.110	**0.000**
Treated	-0.009	-0.038, 0.021	0.564

Supplementary Table S3: Extended participant baseline characteristics

Pt Number	Treatment group	Sex	Age at baseline (years)	Fabry specific therapy	Mutation (or nucleotide change)	eGFR at baseline (mL/min/1.73m2)	Increased left ventricular mass at baseline
1	Untreated	Female	21	Nil	delGlu358	133	No
2	Untreated	Female	51	Nil	delGlu358	87	No
3	Untreated	Female	46	Nil	R227Q	88	No
4	Untreated	Female	61	Nil	c.858_863delinsTTGGG	88	Yes
5	Untreated	Female	20	Nil	N215S	94	No
6	Untreated	Female	23	Nil	N215S	97	No
7	Untreated	Female	42	Nil	N215S	99	No
8	Untreated	Female	21	Nil	c.858_863delinsTTGGG	100	No
9	Untreated	Female	21	Nil	M284T	132	No
10	Untreated	Female	25	Nil	D165Y	98	No
11	Untreated	Female	24	Nil	c1193_1196del	101	No
12	Untreated	Male	41	Nil	N215S	156	No
13	Untreated	Female	38	Nil	R227Q	106	No
14	Untreated	Female	35	Nil	R227Q	122	No
15	Untreated	Female	34	Nil	R227Q	117	No
16	Untreated	Female	71	Nil	Double	86	No
17	Untreated	Female	36	Nil	Double	100	No
18	Untreated	Female	24	Nil	R118C	109	No
19	Untreated	Male	59	Nil	R118C	111	No
20	Untreated	Female	37	Nil	R227Q	75	No
21	Untreated	Male	61	Nil	A143T	64	No
22	Untreated	Female	22	Nil	M284T	108	No
23	Untreated	Female	48	Nil	delA395	93	No
24	Untreated	Female	50	Nil	c.858_863delinsTTGGG	80	No
25	Untreated	Male	22	Nil	E6,c.833dupA	147	No
26	Untreated	Male	52	Nil	N215S	49	No
27	Untreated	Female	46	Nil	delGlu358	106	No
28	Untreated	Female	49	Nil	E6,c.833dupA	127	No
29	Untreated	Female	31	Nil	R227Q	111	No
30	Untreated	Female	78	Nil	c.858_863delinsTTGGG	91	Yes
31	Untreated	Female	59	Nil	M284T	94	No
32	Untreated	Female	38	Nil	N215S	95	No
33	Untreated	Female	60	Nil	N215S	87	No
34	Untreated	Female	31	Nil	pA156T	101	No
35	Untreated	Female	30	Nil	pA156T	103	No
36	Untreated	Female	45	Nil	pA156T	109	No
37	Untreated	Female	70	Nil	N215S	75	No
38	Untreated	Male	38	Nil	N215S	88	No
39	Untreated	Female	44	Nil	M284T	98	No
40	Untreated	Female	18	Nil	M284T	99	No
41	Untreated	Female	28	Nil	M1L	91	No
42	Untreated	Female	28	Nil	M1L	92	No
43	Untreated	Female	57	Nil	N215S	95	No
44	Untreated	Male	29	Nil	N215S	94	No
45	Untreated	Female	27	Nil	G128E	99	No
46	Untreated	Female	39	Nil	R227Q	67	No
47	Untreated	Female	22	Nil	pA156T	122	No
48	Untreated	Female	19	Nil	pA156T	141	No
49	Untreated	Male	21	Nil	E6,c.833dupA	147	No
50	Untreated	Female	37	Nil	N215S	118	No
51	Treated	Male	53	Agalsidase alpha	C52R	49	Yes
52	Treated	Male	17	Agalsidase alpha	c.195-1 G>A	104	No
53	Treated	Female	49	Agalsidase alpha	c.858_863delinsTTGGG	115	No
54	Treated	Male	41	Agalsidase alpha	D165Y	57	Yes
55	Treated	Male	35	Agalsidase beta	W226R		Yes
56	Treated	Female	49	Agalsidase beta	c1193_1196del	17	Yes
57	Treated	Female	58	Agalsidase alpha	N215S	77	Yes
58	Treated	Male	25	Agalsidase alpha	M284T	77	No

59	Treated	Male	69	Agalsidase beta	N215S	97	Yes
60	Treated	Male	37	Agalsidase alpha	Double	127	Yes
61	Treated	Female	60	Agalsidase alpha	R227Q	66	Yes
62	Treated	Male	34	Agalsidase beta	R220X	96	No
63	Treated	Male	36	Agalsidase beta	R220X	94	No
64	Treated	Female	38	Agalsidase beta	delGlu358	77	Yes
65	Treated	Female	58	Agalsidase alpha	delGlu358	64	Yes
66	Treated	Male	30	Agalsidase alpha	R301X	111	No
67	Treated	Female	58	Agalsidase alpha	P205T	87	No
68	Treated	Male	43	Migalastat	L300P	79	Yes
69	Treated	Male	37	Agalsidase beta	W287R	81	Yes
70	Treated	Female	13	Agalsidase alpha	delGlu358	225	No
71	Treated	Male	27	Agalsidase beta	M284T	99	No
72	Treated	Male	51	Agalsidase beta	R301Q	94	Yes
73	Treated	Female	65	Agalsidase beta	Y134S	89	Yes
74	Treated	Male	37	Agalsidase beta	M187T		Yes
75	Treated	Male	31	Agalsidase alpha	M284T	92	Yes
76	Treated	Female	61	Agalsidase alpha	N215S	71	Yes
77	Treated	Male	21	Agalsidase alpha	N215S		No
78	Treated	Female	48	Agalsidase beta	G128E	63	Yes
79	Treated	Male	29	Agalsidase alpha	G128E	100	No
80	Treated	Male	39	Agalsidase beta	G128E	71	Yes
81	Treated	Female	46	Agalsidase alpha	M1L	78	Yes
82	Treated	Male	47	Agalsidase beta	M1L		Yes
83	Treated	Male	39	Agalsidase beta	M284T	55	Yes
84	Treated	Female	42	Agalsidase alpha	M284T	75	Yes
85	Treated	Male	30	Agalsidase alpha	M284T	14	Yes
86	Treated	Male	31	Agalsidase alpha	G128E	110	No
87	Treated	Male	34	Agalsidase beta	G128E	92	No
88	Treated	Male	52	Agalsidase beta	pA156T	59	Yes

Supplementary Table S4: Post-hoc exploratory analysis by participant sex

	Group by time interaction from linear mixed effects model*		
	β-coefficient	95% confidence interval	P-value
**Overall (n=88)**			
Lumbar spine Z-score	-0.026	-0.078, 0.027	0.334
Total hip Z-score	-0.110	-0.158, -0.061	**<0.001**
Femoral neck Z-score	-0.090	-0.142, -0.038	**0.001**
**Female patients (n=55)**			
Lumbar spine Z-score	-0.067	-0.134, -0.001	**0.046**
Total hip Z-score	-0.123	-0.201, -0.046	**0.002**
Femoral neck Z-score	-0.115	-0.201, -0.030	**0.008**
**Male patients (n=33)**			
Lumbar spine Z-score	-0.015	-0.118, -0.089	0.784
Total hip Z-score	-0.086	-0.154, -0.017	**0.014**
Femoral neck Z-score	-0.067	-0.135, 0.000	0.050

Table shows linear mixed effects model beta coefficient for group-by-time change in Z-score (for lumbar spine, total hip and femoral neck bone density). All models have been adjusted for baseline values of age, sex, body mass index, kidney function (estimated glomerular filtration rate), use of anti-epileptic medications and smoking history. Overall model shown for all participants, as well as exploratory analyses where models were fitted after restriction to either female or male participants.
